# The Annual American Men's Internet Survey of Behaviors of Men Who Have Sex With Men in the United States: 2016 Key Indicators Report

**DOI:** 10.2196/11313

**Published:** 2019-02-20

**Authors:** Maria Zlotorzynska, Patrick Sullivan, Travis Sanchez

**Affiliations:** 1 Department of Epidemiology Rollins School of Public Health Emory University Atlanta, GA United States

**Keywords:** bisexual, gay, HIV, homosexual, internet, MSM, STD, surveillance, survey

## Abstract

The American Men’s Internet Survey (AMIS) is an annual Web-based behavioral survey of men who have sex with men (MSM) living in the United States. This Rapid Surveillance Report describes the fourth cycle of data collection (September 2016 through February 2017; AMIS 2016). The key indicators are the same as previously reported for AMIS (December 2013 through May 2014, AMIS 2013; November 2014 through April 2015, AMIS 2014; and September 2015 through April 2016, AMIS 2015). The AMIS survey methodology has not substantively changed since AMIS 2015. MSM were recruited from a variety of websites using banner advertisements and email blasts. Additionally, participants from AMIS 2015 who agreed to be recontacted for future research were emailed a link to the AMIS 2016 survey. Men were eligible to participate if they were ≥15 years old, resided in the United States, provided a valid US zone improvement plan code, and reported ever having sex with a man or identified as gay or bisexual. We examined demographic and recruitment characteristics using multivariable regression modeling (*P*<.05) stratified by participants’ self-reported HIV status. The AMIS 2016 round of data collection resulted in 10,166 completed surveys from MSM representing every US state, Puerto Rico, Guam, and the US Virgin Islands. Participants were mainly non-Hispanic white, over the age of 40 years, living in the Southern United States and urban areas, and recruited from general social networking websites. Self-reported HIV prevalence was 10.80% (1098/10,166). Compared to HIV-negative/unknown-status participants, HIV-positive participants were more likely to have had anal sex without a condom with a male partner in the past 12 months (75.77% vs 65.88%, *P*<.001) and more likely to have had anal sex without a condom with a serodiscordant or unknown-status partner (33.24% vs 16.06%, *P*<.001). The reported use of marijuana, methamphetamines, and other illicit substances in the past 12 months was higher among HIV-positive participants than among HIV-negative/unknown-status participants (28.05% vs 24.99%, 11.48% vs 2.16%, and 27.60% vs 18.22%, respectively; all *P*<.001). Most HIV-negative/unknown-status participants (79.93%, 7248/9068) reported ever having a previous HIV test, and 56.45% (5119/9068) reported undergoing HIV testing in the past 12 months. HIV-positive participants were more likely to report testing and diagnosis of sexually transmitted infections than HIV-negative/unknown-status participants (70.86% vs 40.13% and 24.04% vs 8.97%, respectively; both *P*<.001).

## Introduction

The American Men’s Internet Survey (AMIS) is an annual online behavioral survey of men who have sex with men (MSM), living in the United States. AMIS was developed to produce timely data from large-scale monitoring of behavior trends among MSM recruited online. It was designed to complement the Centers for Disease Control and Prevention’s National HIV Behavioral Surveillance (NHBS) system, which collects data on MSM in major US cities every 3 years through venue-based recruitment [1]. The methods and previous AMIS cycle data (AMIS 2013, AMIS 2014, and AMIS 2015) have been previously published [2-4].

This supplemental report updates previous information with data collected in AMIS 2016. Methods in the AMIS 2016 did not change from the previously published methods, unless otherwise noted. An in-depth analysis and discussion of multiyear trends for indicators reported herein has been published and includes data for the first four cycles of AMIS (AMIS 2013 through AMIS 2016) [5].

## Methods

### Recruitment and Enrollment

Similar to the prior year’s recruitment process, AMIS participants were recruited through convenience sampling from a variety of websites using banner advertisements or email blasts to website members (hereafter referred to generically as “ads”). For AMIS 2016, data were collected from September 2016 through February 2017. The survey was not incentivized. Data on the number of clicks on all banner ads were obtained directly from the websites. Men who clicked on the ads were taken directly to the survey website hosted on a secure server administered by SurveyGizmo (Boulder, Colorado). Participants were also recruited by emailing participants from the previous cycle of AMIS (AMIS 2015) who consented to be recontacted for future studies. To be eligible for the survey, participants had to be ≥15 years of age, consider themselves as male, reside in the United States, and report that they either had oral or anal sex with a man at least once in the past or identify as gay or bisexual (hereafter referred to as men who have sex with men [MSM]). Persons who were <15 years of age or refused to provide their age were not asked any other screening questions. MSM who met the eligibility criteria and consented to participate in the study started the online survey immediately. The full questionnaire for AMIS 2016 is presented in Multimedia Appendix 1.

Several data-cleaning steps were performed on the raw dataset of eligible responses to obtain the final analysis dataset. First, deduplication of survey responses was performed in the same manner as in previous AMIS cycles [2-5]. Briefly, the demographic data for near-complete (>70%) survey responses with nonunique internet protocol addresses were compared, and responses that showed 100% match for all characteristics were considered to be duplicate responses. Only the observation with the highest survey completion was retained. The dataset was then limited to those surveys deemed successful (ie, observations with no missing values for the first question of at least two consecutive sections). Finally, the dataset was restricted to include participants who reported having oral or anal sex in the past 12 months and provided a valid US zone improvement plan (ZIP) code. ZIP codes were validated in same manner as in AMIS 2015 [4]. Valid US ZIP codes were those that could be matched to the ZIP code for county crosswalk files created by the US Department of Housing and Urban Development [6]. Any ZIP codes that could not be matched to this list were then hand validated by checking against the ZIP code locator tool on the US Postal Service website [7]. ZIP codes that could not be found were classified as invalid.

### Measures and Analyses

For AMIS 2016 analyses, participants were categorized as either AMIS 2015 participants who took the survey again or new participants from the website/app based on target audience and purpose: gay social networking (n=2), gay general interest (n=1), general social networking (n=3), and geospatial social networking (n=2). Recruitment outcomes and demographic characteristics for the AMIS 2015 participants are presented in Tables 1 and 2, and thereafter, they are recategorized according to their original source of recruitment. We do not provide the names of the websites/apps to preserve operator and client privacy, particularly when a category has only one operator. Participants whose data were eligible, unduplicated, and successful and who provided consent, reported male-male sex in the past 12 months, and provided a valid US ZIP code were included in analyses of participant characteristics and behavior.

To facilitate comparisons, the key indicators and analytic approach used in AMIS were designed to mirror those used by the NHBS system [8]. Population density was defined in the same manner as in AMIS 2015 and was based on the National Center for Health Statistics Rural-Urban classification scheme for counties [9]. The self-reported HIV status was categorized as HIV positive or HIV negative/unknown status, consistent with surveillance reports produced by the NHBS system [8]. Three substance use behaviors in the past 12 months were assessed: use of nonprescribed marijuana, use of methamphetamines, and use of any illicit drug other than marijuana or methamphetamines. All other indicators assessed remained unchanged from AMIS 2015 [4].

The analysis methods for AMIS 2016 did not substantively differ from those previously published but are repeated in this report for clarity. Overall, chi-square tests were used to identify whether participant characteristics differed significantly between recruitment sources. Multivariable logistic regression modeling was used to determine significant differences in behaviors based on the self-reported HIV status while controlling for race/ethnicity, age group, NHBS city residency, and type of recruitment website. Metropolitan statistical areas included in the NHBS system in 2016 were Atlanta, GA; Baltimore, MD; Boston, MA; Chicago, IL; Dallas, TX; Denver, CO; Detroit, MI; Houston, TX; Los Angeles, CA; Miami, FL; Nassau-Suffolk, NY; New Orleans, LA, New York City, NY; Newark, NJ; Philadelphia, PA; San Diego, CA; San Francisco, CA; San Juan, PR; Seattle, WA; and Washington, DC. HIV testing behaviors were only examined among those who did not report that they were HIV positive, and these data were presented by participant characteristics. Multivariable logistic regression results are presented as Wald chi-square *P* values to denote an independently significant difference in the behavior for each subgroup compared to a reference group. Statistical significance was set at *P*<.05.

**Table 1:** Recruitment outcomes for the American Men’s Internet Survey, United States, 2016.

Recruitment outcomes			Total		Gay social networking (n=2)		General gay interest (n=1)		General social networking (n=3)		Geospatial social networking (n=2)		AMIS^a^ 2015 participants
Clicked ad (N)			147,143		4162		557		58,917		83,507		Not applicable
Screened^b^, n (%)			51,876 (35.26)		2877 (69.13)		181 (32.50)		39,281 (66.67)		8137 (9.74)		1400
**Ineligible^c^, n (%)**			23,173 (44.67)		564 (19.60)		147 (81.22)		19,271 (49.06)		3039 (37.35)		152 (10.86)
	Not ≥15 years of age^d^	16,643 (71.82)		438 (77.66)		91 (61.90)		13,572 (70.43)		2441 (80.32)		101 (66.45)	
	Not male^d^	19,079 (82.33)		511 (90.60)		94 (63.95)		15,641 (81.16)		2704 (88.98)		129 (84.87)	
	Not MSM^e^ ever or not identifying as gay/bisexual^d^	22,282 (96.16)		549 (97.34)		99 (67.35)		18,721 (97.15)		2790 (91.81)		123 (80.92)	
	Nonresident^d^	18,989 (81.94)		471 (83.51)		144 (97.96)		15,383 (79.82)		2845 (93.62)		146 (96.05)	
Eligible^c^, n (%)			28,703 (55.33)		2313 (80.40)		34 (18.78)		20,010 (50.94)		5098 (62.65)		1248 (89.14)
Consented^f^, n (%)			20,583 (71.71)		1716 (74.19)		28 (82.35)		13,776 (68.85)		3928 (77.05)		1135 (90.95)
Unduplicated^g^, n (%)			18,038 (87.64)		1604 (93.47)		23 (82.14)		11,876 (86.21)		3501 (89.13)		1034 (91.10)
Success^h^, n (%)			11,636 (64.51)		1242 (77.43)		14 (60.87)		7594 (63.94)		1870 (53.41)		916 (88.59)
MSM in past 12 months^i^, n (%)			10,222 (87.85)		1165 (93.80)		13 (92.86)		6443 (84.84)		1756 (93.90)		845 (92.25)
Valid ZIP^j^ code^k^, n (%)			10,166 (99.45)		1160 (99.57)		13 (100.00)		6401 (99.35)		1750 (99.66)		842 (99.64)

^a^AMIS: American Men’s Internet Survey.

^b^Proportion of total participants who clicked on the ad, including those who started the screening questionnaire.

^c^Proportion of total participants screened. Participants who did not complete the screening questionnaire were considered ineligible.

^d^Proportion of total ineligible participants, including those who did not respond to the question.

^e^MSM: men who have sex with men.

^f^Proportion of eligible participants.

^g^Proportion of participants who consented. Deduplication removes participants who were marked as duplicates using the internet protocol address and demographic data matching.

^h^Proportion of unduplicated participants. Success in deduplication removes participants who did not pass the test for survey completeness.

^i^Proportion of successes.

^j^ZIP: zone improvement plan.

^k^Proportion of men who had sex with men in the past 12 months. Valid US ZIP codes were those that could be matched to the ZIP code for county crosswalk files created by the US Department of Housing and Urban Development. Any ZIP codes that could not be matched to this list were then hand validated by checking against the ZIP code-locator tool on the US Postal Service website. ZIP codes that could not be found were classified as invalid.

**Table 2:** Characteristics of men who have sex with men in the American Men's Internet Survey by recruitment type, United States, 2016.

Participant characteristics		Recruitment type											*P* value^b^
		Total		Gay social networking (n=2)		General gay interest (n=1)		General social networking (n=3)		Geospatial social networking (n=2)		AMIS^a^ 2015 participants	
**Race/ethnicity, n (%)**													<.001
	Black, non-Hispanic	879 (8.65)	35 (3.02)		0 (0.00)		664 (10.37)		141 (8.06)		39 (4.63)		
	Hispanic	1311 (12.90)	73 (6.29)		2 (15.38)		852 (13.31)		286 (16.34)		98 (11.64)		
	White, non-Hispanic	7073 (69.58)	986 (85.00)		8 (61.54)		4274 (66.77)		1157 (66.11)		648 (76.96)		
	Other or multiple races	903 (8.88)	66 (5.69)		3 (23.08)		611 (9.55)		166 (9.49)		57 (6.77)		
**Age (years), n (%)**													<.001
	15-24	2718 (26.74)	27 (2.33)		6 (46)		2268 (35.43)		271 (15.49)		146 (17.34)		
	25-29	1693 (16.65)	34 (2.93)		2 (15)		1223 (19.11)		265 (15.14)		169 (20.07)		
	30-39	1414 (13.91)	92 (7.93)		1 (8)		719 (11.23)		455 (26.00)		147 (17.46)		
	≥40	4341 (42.70)	1007 (86.81)		4 (31)		2191 (34.23)		759 (43.37)		380 (45.13)		
**Region, n (%)**													<.001
	Northeast	1879 (18.48)	235 (20.26)		2 (15.38)		1185 (18.51)		306 (17.49)		151 (17.93)		
	Midwest	1988 (19.56)	232 (20.00)		4 (30.77)		1352 (21.12)		233 (13.31)		167 (19.83)		
	South	4055 (39.89)	422 (36.38)		4 (30.77)		2506 (39.15)		800 (45.71)		323 (38.36)		
	West	2240 (22.03)	271 (23.36)		3 (23.08)		1355 (21.17)		410 (23.43)		201 (23.87)		
	US-dependent areas	4 (0.04)	0 (0)		0 (0)		3 (0.05)		1 (0.06)		0 (0)		
**NHBS^c^ city resident, n (%)**													<.001
	Yes	4224 (41.55)	500 (43.10		7 (53.85)		2291 (35.81)		1085 (62.00)		341 (40.50)		
	No	5942 (58.45)	660 (56.90		6 (46.15)		4110 (64.21)		665 (38.02)		501 (59.50)		
**Population density^d^, n (%)**													<.001
	Urban	4288 (42.18)	436 (37.59)		5 (38.46)		2538 (39.65)		944 (53.94)		365 (43.35)		
	Suburban	2200 (21.64)	316 (27.24)		6 (46.15)		1298 (20.28)		409 (23.37)		171 (20.31)		
	Small/medium metropolitan	2790 (27.44)	305 (26.29)		1 (7.69)		1929 (30.14)		305 (17.43)		250 (29.69)		
	Rural	884 (8.70)	103 (8.88)		1 (7.69)		633 (9.89)		91 (5.20)		56 (6.65)		
**Self-reported HIV status, n (%)**													<.001
	Positive	1098 (10.80)	125 (10.78)		2 (15.38)		619 (9.67)		263 (15.03)		89 (10.57)		
	Negative	7089 (69.73)	828 (71.38)		9 (69.23)		4283 (66.91)		1291 (73.77)		678 (80.52)		
	Unknown	1979 (19.47)	207 (17.84)		2 (15.38)		1499 (23.42)		196 (11.20)		75 (8.91)		
Total (N)		10,166	1160		13		6401		1750		842		

^a^AMIS: American Men’s Internet Survey.

^b^Chi-square test for difference in characteristics between recruitment types.

^c^NHBS: National HIV Behavioral Surveillance.

^d^The National Center for Health Statistics urban/rural category could not be assigned for four participants living in US territories.

## Results

AMIS 2016 was performed from September 2016 through February 2017 and resulted in 147,143 persons clicking on the ads and landing on the study’s recruitment page (Table 1). Most persons who clicked on the ads were from geospatial networking websites (83,507/147,143; 56.75%). Of the 4513 participants who completed the AMIS 2015 survey and were emailed links to the AMIS 2016 survey, 31.02% (1400) clicked on the link. Just over one-third (35.26%) of all of participants who landed on the study page started the screening process and 55.33% of them were eligible. The most common reason for ineligibility was not ever having male-male sex or not identifying as gay or bisexual. Almost three-quarters (71.71%) of participants who were eligible consented to participate in the survey. A total of 2545 (12.36%) surveys were likely from duplicate participants. Among unduplicated surveys, almost two-thirds (64.51%) were considered successful. Most successful surveys were from men who reported having sex with another man in the past 12 months (87.85%). Almost all these surveys (10,166/10,222; 99.45%) provided a valid US ZIP code. Overall, the completion rate was 6.9%, with an analytical sample consisting of 10,166 surveys from 147,143 clicks.

**Figure 1:**
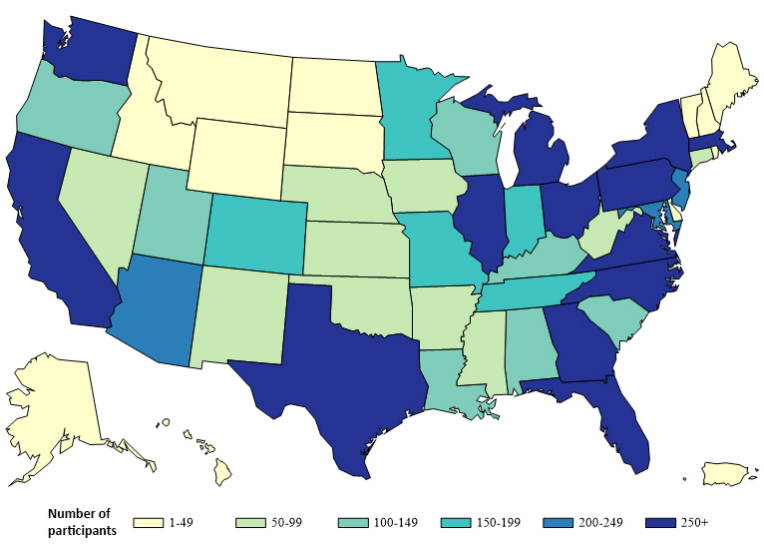
Number of men who have sex with men who participated in the American Men’s Internet Survey by state, 2016.

Over two-thirds (7073/10,166; 69.58%) of the participants included in this report were non-Hispanic white, and less than half were ≥40 years of age (4341/10,166; 42.70%); the most common region of residence was the South followed by the West (Table 2). Participants were recruited from all US states, and there were at least 100 participants from each of the 28 states and the District of Columbia (Figure 1). About 4 in 10 (4224/10,166; 41.55%) participants resided in an NHBS city and about the same proportion (4288/10,166; 42.18%) lived in an urban county. Overall, 10.80% (1098/10,166) of participants were HIV positive, 69.73% were HIV negative (7089/10,166), and 19.74% (1979/10,166) had an unknown HIV status. All participant characteristics differed significantly based on the recruitment source (Table 2).

Most participants reported having anal sex without a condom with another man in the past 12 months (Table 3). Compared to HIV-negative/unknown-status participants, those who were HIV positive were significantly more likely to report anal intercourse without a condom (adjusted odds ratio [aOR]=1.79, 95% CI: 1.53-2.08), including with male partners who were of discordant or unknown status (aOR=2.76, 95% CI: 2.38-3.19). Stratified by the serostatus group, anal intercourse without a condom differed significantly by race/ethnicity (HIV-negative/unknown-status participants only), age group (HIV-negative/unknown-status participants only), and recruitment website (HIV-positive and HIV-negative/unknown-status participants). Anal intercourse without a condom with partners of discordant or unknown HIV status differed significantly by race/ethnicity, age, and recruitment website for both HIV-positive participants and HIV-negative/unknown-status participants.

**Table 3:** Sexual Behaviors with male partners of men who have sex with men in the American Men's Internet Survey, United States, 2016.

Participant characteristics			Participants (N)	Sexual behaviors with male partners in the past 12 months			
				Anal intercourse without a condom		Anal intercourse without a condom with a partner of discordant or unknown HIV status	
				n (%)	*P* value^a^	n (%)	*P* value^a^
**HIV positive**			1098	832 (75.77)	<.001^b^	365 (33.24)	<.001^b^
	**Race/ethnicity**						
		Black, non-Hispanic	248	175 (70.56)	.17	76 (30.65)	.70
		Hispanic	130	96 (73.85)	.23	34 (26.15)	.001
		White, non-Hispanic	632	492 (77.85)	Ref^a^	218 (34.49)	Ref^a^
		Other or multiple races	88	69 (78.41)	.34	37 (42.05)	.05
	**Age (years)**						
		15-24	59	50 (84.75)	.37	21 (35.59)	.87
		25-29	97	80 (82.47)	.78	48 (49.48)	.008
		30-39	186	159 (85.48)	.16	76 (40.86)	.41
		≥40	756	543 (71.83)	Ref^a^	220 (29.10)	Ref^a^
	**NHBS^c^** **city resident**						
		Yes	568	429 (75.53)	.78	195 (34.33)	.43
		No	530	403 (76.04)	Ref^a^	170 (32.08)	Ref^a^
	**Recruitment type**						
		Gay social networking	139	102 (73.38)	.45	58 (41.73)	.004
		General gay interest^d^	<5	N/A^e^	N/A	N/A	N/A
		General social networking	651	471 (72.35)	Ref^a^	193 (29.65)	Ref^a^
		Geospatial social networking	303	255 (84.16)	.007	111 (36.63)	.47
**HIV negative or unknown status**			9068	5974 (65.88)	Ref^a^	1456 (16.06)	Ref^a^
	**Race/ethnicity**						
		Black, non-Hispanic	631	388 (61.49)	.002	116 (18.38)	.08
		Hispanic	1181	833 (70.53)	<.001	218 (18.46)	.12
		White, non-Hispanic	6441	4231 (65.69)	Ref^a^	1010 (15.68)	Ref^a^
		Other or multiple races	815	522 (64.05)	.28	112 (13.74)	.009
	**Age (years)**						
		15-24	2659	1694 (63.71)	<.001	417 (15.68)	.87
		25-29	1596	1202 (75.31)	<.001	285 (17.86)	.03
		30-39	1228	892 (72.64)	.002	232 (18.89)	.09
		≥40	3585	2186 (60.98)	Ref^a^	522 (14.56)	Ref^a^
	**NHBS^c^** **city resident**						
		Yes	3656	2436 (66.63)	>.99	630 (17.23)	.14
		No	5412	3538 (65.37)	Ref^a^	826 (15.26)	Ref^a^
	**Recruitment type**						
		Gay social networking	1119	626 (55.94)	<.001	210 (18.77)	.007
		General gay interest	73	50 (68.49)	.76	10 (13.70)	.38
		General social networking	6125	4026 (65.73)	Ref^a^	911 (14.87)	Ref^a^
		Geospatial social networking	1731	1257 (72.62)	<.001	321 (18.54)	.35

^a^Wald chi-square from multivariate logistic regression comparing behavior (yes vs no) between a group with specific characteristics and a reference group (Ref).

^b^Wald chi-square from multivariate logistic regression comparing behavior (yes vs no) among HIV-positive participants and HIV-negative or unknown-serostatus participants. Model controlled for race/ethnicity, age, National HIV Behavioral Surveillance system residency, and recruitment type.

^C^NHBS: National HIV Behavioral Surveillance.

^d^To prevent identification, data for groups with less than five participants are not presented.

^e^N/A: not applicable.

Over one-quarter (308/1098; 28.05%) of HIV-positive participants reported using marijuana in the past 12 months (Table 4). Compared to HIV-negative/unknown-status participants, HIV-positive participants were significantly more likely to report use of marijuana (aOR=1.63, 95% CI: 1.40-1.90), methamphetamines (aOR=5.53, 95% CI: 4.30-7.11), and other illicit substances in the past 12 months (aOR=2.15, 95% CI: 1.84-2.52). Among HIV-positive participants, the use of marijuana did not vary significantly for any participant characteristic, but the use of methamphetamines varied significantly by race/ethnicity, age, and recruitment site. In this group, the use of other illicit substances varied significantly by race/ethnicity, age, residence in an NHBS city, and recruitment site. Use of marijuana, methamphetamines, and other illicit substances differed significantly by race/ethnicity and age among HIV-negative/unknown-status participants. Additionally, the use of marijuana and other illicit substances differed significantly by residence in an NHBS city, and the use of other illicit substances differed significantly by recruitment site among HIV-negative/unknown-status participants.

HIV testing behaviors were examined among participants who were not HIV positive (Table 5). Most participants (7248/9068; 79.93%) were previously tested for HIV infection, and just over half (5119/9068; 56.45%) were tested in the past 12 months. HIV testing behavior, both ever tested and tested in the past 12 months, differed significantly by race/ethnicity, age, residence in an NHBS city, and type of recruitment website.

Compared to HIV-negative/unknown-status participants, HIV-positive participants were significantly more likely to report testing for sexually transmitted infection (STI) (aOR=3.62, 95% CI: 3.13-4.19) and STI diagnosis (aOR=3.25, 95% CI: 2.74-3.87) in the past 12 months (Table 6). The most common STI diagnosis among HIV-positive participants was syphilis (164/1098; 14.94%), followed by gonorrhea (125/1098; 11.38%) and chlamydia (108/1098; 9.84%). Gonorrhea was the most common STI diagnosis among HIV-negative/unknown-status participants (456/9068; 5.03%), followed by chlamydia (412/9068; 4.54%) and syphilis (226/9068; 2.49%). Among HIV-negative/unknown-status participants, STI testing differed significantly by race/ethnicity and age. Among both HIV-positive and HIV-negative/unknown-status participants, STI testing differed significantly by NHBS city residence and type of recruitment website, and STI diagnosis differed significantly by age, NHBS city residence, and type of recruitment website.

**Table 4:** Substance use behaviors of men who have sex with men in the American Men's Internet Survey, United States, 2016.

Participant characteristics			Participants (N)		Substance use behaviors in the past 12 months												
					Used marijuana				Used methamphetamines				Used other substance(s)				
					n (%)		*P* value^a^		n (%)		*P* value^a^			n (%)		*P* value^a^	
**HIV positive**			1098		308 (28.05)		<.001^b^		126 (11.48)		<.001^b^			303 (27.60)		<.001^b^	
	**Race/ethnicity**																
		Black, non-Hispanic		248		66 (26.61)		.52		16 (6.45)		.02			52 (20.97)		.04
		Hispanic		130		40 (30.77)		.98		22 (16.92)		.12			44 (33.85)		.21
		White, non-Hispanic		632		176 (27.85)		Ref^a^		75 (11.87)		Ref^a^			183 (28.96)		Ref^a^
		Other or multiple races		88		26 (29.55)		.96		13 (14.77)		.45			24 (27.27)		.64
	**Age (years)**																
		15-24		59		22 (37.29)		.29		5 (8.47)		.51			21 (35.59)		.60
		25-29		97		33 (34.02)		.80		10 (10.31)		.72			41 (42.27)		.03
		30-39		186		71 (38.17)		.18		33 (17.74)		.03			65 (34.95)		.96
		≥40		756		182 (24.07)		Ref^a^		78 (10.32)		Ref^a^			176 (23.28)		Ref^a^
	**NHBS^c^** **city resident**																
		Yes		568		174 (30.63)		.10		78 (13.73)		.11			175 (30.81)		.04
		No		530		134 (25.28)		Ref^a^		48 (9.06)		Ref^a^			128 (24.15)		Ref^a^
	**Recruitment type**																
		Gay social networking		139		36 (25.90)		.98		17 (12.23)		.93			34 (24.46)		.56
		General gay interest^d^		<5		N/A^e^		N/A		N/A		N/A			N/A		N/A
		General social networking		651		170 (26.11)		Ref^a^		50 (7.68)		Ref^a^			153 (23.50)		Ref^a^
		Geospatial social networking		303		100 (33.00)		.35		57 (18.81)		.007			113 (37.29)		.01
**HIV negative or unknown status**			9068		2266 (24.99)		Ref^a^		196 (2.16)		Ref^a^			1652 (18.22)		Ref^a^	
	**Race/ethnicity**																
		Black, non-Hispanic		631		126 (19.97)		.006		8 (1.27)		.049			86 (13.63)		.001
		Hispanic		1181		344 (29.13)		.04		37 (3.13)		.03			275 (23.29)		<.001
		White, non-Hispanic		6441		1597 (24.79)		Ref^a^		128 (1.99)		Ref^a^			1152 (17.89)		Ref^a^
		Other or multiple races		815		199 (24.42)		.33		23 (2.82)		.12			139 (17.06)		.27
	**Age (years)**																
		15-24		2659		886 (33.32)		<.001		37 (1.39)		.03			548 (20.61)		.001
		25-29		1596		500 (31.33)		<.001		35 (2.19)		.60			388 (24.31)		.18
		30-39		1228		322 (26.22)		.74		42 (3.42)		.04			270 (21.99)		<.001
		≥40		3585		558 (15.56)		Ref^a^		82 (2.29)		Ref^a^			446 (12.44)		Ref^a^
	**NHBS^c^** **city resident**																
		Yes		3656		1024 (28.01)		<.001		94 (2.57)		.48			779 (21.31)		<.001
		No		5412		1242 (22.95)		Ref^a^		102 (1.88)		Ref^a^			873 (16.13)		Ref^a^
	**Recruitment type**																
		Gay social networking		1119		201 (17.96)		.71		37 (3.31)		.13			150 (13.40)		.68
		General gay interest		73		16 (21.92)		.55		1 (6.25)		.48			12 (16.44)		.52
		General social networking		6125		1572 (25.67)		Ref^a^		90 (1.47)		Ref^a^			1068 (17.44)		Ref^a^
		Geospatial social networking		1731		473 (27.33)		.11		68 (3.93)		.08			419 (24.21)		<.001

^a^Wald chi-square from multivariable logistic regression comparing behavior (yes vs no) between groups with specific characteristics and a reference (Ref) group.

^b^Wald chi-square from multivariable logistic regression comparing behavior (yes vs no) between HIV-positive participants and HIV-negative or unknown-serostatus participants. Model controlled for race/ethnicity, age, National HIV Behavioral Surveillance system residency, and website type.

^c^NHBS: National HIV Behavioral Surveillance.

^d^To prevent identification, data for groups with less than five participants are not presented.

^e^N/A: not applicable.

**Table 5:** HIV testing behaviors of HIV-negative or unknown-status men who have sex with men in the American Men's Internet Survey, United States, 2016.

Participant characteristics		Participants (N)	HIV testing behaviors			
			HIV tested ever		HIV tested in past 12 months	
			n (%)	*P* value^a^	n (%)	*P* value^a^
**Race/ethnicity**						
	Black, non-Hispanic	631	563 (89.22)	<.001	440 (69.73)	<.001
	Hispanic	1181	916 (77.56)	.09	710 (60.12)	.68
	White, non-Hispanic	6441	5134 (79.71)	Ref^a^	3512 (54.53)	Ref^a^
	Other or multiple races	815	635 (77.91)	.30	457 (56.07)	.02
**Age (years)**						
	15-24	2659	1521 (57.20)	<.001	1207 (45.39)	<.001
	25-29	1596	1393 (87.28)	.004	1040 (65.16)	<.001
	30-39	1228	1129 (91.94)	<.001	829 (67.51)	<.001
	≥40	3585	3205 (89.40)	Ref^a^	2043 (56.99)	Ref^a^
**NHBS^b^** **city resident**						
	Yes	3656	3112 (85.12)	<.001	2356 (64.44)	<.001
	No	5412	4136 (76.42)	Ref^a^	2763 (51.05)	Ref^a^
**Recruitment type**		1119	926 (82.75)			
	Gay social networking	73	66 (90.41)	<.001	577 (51.56)	<.001
	General gay interest	6125	4676 (76.34)	.18	44 (60.27)	.93
	General social networking	1731	1563 (90.29)	Ref^a^	3193 (52.13)	Ref^a^
	Geospatial social networking	1119	926 (82.75)	<.001	1292 (74.64)	<.001
Total		9068	7248 (79.93)		5119 (56.45)	

^a^Wald chi-square from multivariable logistic regression comparing behavior (yes vs no) between groups with specific characteristics and a reference (Ref) group.

^b^NHBS: National HIV Behavioral Surveillance.

**Table 6:** Sexually transmitted infection testing and diagnosis of men who have sex with men participants in the American Men's Internet Survey, United States, 2016.

Participant characteristics			Participants (N)	STI^a^ history in the past 12 months				
				Tested for any STI		Diagnosed with any STI		
				n (%)	*P* value^b^		n (%)	*P* value^b^
**HIV positive**			1098	778 (70.86)	<.001^c^		264 (24.04)	<.001^c^
	**Race/ethnicity**							
		Black, non-Hispanic	248	178 (71.77)	.40		67 (27.02)	.30
		Hispanic	130	105 (80.77)	.24		39 (30.00)	.82
		White, non-Hispanic	632	426 (67.41)	Ref^b^		134 (21.20)	Ref^b^
		Other or multiple races	88	69 (78.41)	.40		24 (27.27)	.94
	**Age (years)**							
		15-24	59	47 (79.66)	.52		16 (27.12)	.90
		25-29	97	80 (82.47)	.28		37 (38.14)	.03
		30-39	186	152 (81.72)	.45		67 (36.02)	.26
		≥40	756	499 (66.01)	Ref^b^		144 (19.05)	Ref^b^
	**NHBS^d^** **city resident**							
		Yes	568	426 (75.00)	.03		166 (29.23)	.004
		No	530	352 (66.42)	Ref^b^		98 (18.49)	Ref^b^
	**Recruitment website type**							
		Gay social networking	139	92 (66.19)	.44		29 (20.86)	.90
		General gay interest^e^	<5	N/A^f^	N/A		N/A	N/A
		General social networking	651	439 (67.43)	Ref^b^		124 (19.05)	Ref^b^
		Geospatial social networking	303	242 (79.87)	.004		110 (36.30)	<.001
**HIV negative or unknown status**			9068	3639 (40.13)	Ref^b^		813 (8.97)	Ref^b^
	**Race/ethnicity**							
		Black, non-Hispanic	631	313 (49.60)	.01		77 (12.20)	.20
		Hispanic	1181	566 (47.93)	.16		154 (13.04)	.08
		White, non-Hispanic	6441	2409 (37.40)	Ref^b^		498 (7.73)	Ref^b^
		Other or multiple races	815	351 (43.07)	.35		84 (10.31)	.59
	**Age (years)**							
		15-24	2659	935 (35.16)	<.001		216 (8.12)	.21
		25-29	1596	848 (53.13)	<.001		213 (13.35)	<.001
		30-39	1228	617 (50.24)	.01		155 (12.62)	.23
		≥40	3585	1239 (34.56)	Ref^b^		229 (6.39)	Ref^b^
	**NHBS^d^** **city resident**							
		Yes	3656	1762 (48.19)	<.001		438 (11.98)	<.001
		No	5412	1877 (34.68)	Ref^b^		375 (6.93)	Ref^b^
	**Recruitment website type**							
		Gay social networking	1119	338 (30.21)	<.001		55 (4.92)	.05
		General gay interest	73	27 (36.99)	.35		5 (6.85)	.53
		General social networking	6125	2264 (36.96)	Ref^b^		469 (7.66)	Ref^b^
		Geospatial social networking	1731	1001 (57.83)	<.001		284 (16.41)	<.001

^a^STI: sexually transmitted infection and includes chlamydia, gonorrhea, and syphilis.

^b^Wald chi-square from multivariable logistic regression comparing behavior (yes vs no) between groups with specific characteristics and a reference (Ref) group.

^c^Wald chi-square from multivariable logistic regression comparing behavior (yes vs no) between HIV-positive participants and HIV-negative or unknown-serostatus participants. Model controlled for race/ethnicity, age, National HIV Behavioral Surveillance system residency, and website type.

^d^NHBS: National HIV Behavioral Surveillance.

^e^To prevent identification, data for groups with less than five participants are not presented.

^f^N/A: not applicable.

## Appendix

Multimedia Appendix 1 American Men’s Internet Survey 2016 questionnaire.

## Abbreviations

aOR: adjusted odds ratio
AMIS: American Men’s Internet Survey
MSM: men who have sex with men
NHBS: National HIV Behavioral Surveillance
STI: sexually transmitted infection
ZIP: zone improvement plan
