# Supplementary material for: The Annual American Men's Internet Survey of Behaviors of Men Who Have Sex With Men in the United States: 2016 Key Indicators Report
Source: JMIR Public Health Surveill. 2019 Feb 20;5(1):e11313. doi: 10.2196/11313 (PMC6401665; doi:10.2196/11313)
Supplement: Multimedia Appendix 1 [file publichealth_v5i1e11313_app1.pdf]

American Men's Internet Survey (AMIS) 2016:

Online HIV Behavioral Survey of Men Who Have Sex with Men

EMORY
ROLLINS SCHOOL OF PUBLIC HEALTH

For data requests and additional information:

Email: amis@emory.edu

Phone: 404-727-8799

Address: PRISM Health
1518 Clifton Road NE
Atlanta, Georgia 30322

Sex is the Question - 2016

Landing page

Join over 10,000 men in the U.S. by taking our annual sexual health survey!

"Sex is the Question" collects information to help researchers better understand patterns of behavior – both sexual and health promoting – among men and to help ensure prevention resources have the greatest impact for the community. Click or tap the arrow or "Next" button below to be a part of this national effort!

Eligibility Screener

Questions marked with * are required.

How old are you?*

____

Since September 2016, have you already completed at least part of Sex is the Question?

( ) No
( ) Yes
( ) I prefer not to answer
( ) Don't know

Do you consider yourself to be Hispanic or Latino?*

( ) No
( ) Yes
( ) I prefer not to answer
( ) Don't know

Which racial group or groups do you consider yourself to be in? Check all that apply.*

[ ] American Indian or Alaska Native
[ ] Asian
[ ] Black or African American
[ ] Native Hawaiian or Other Pacific Islander
[ ] White
[ ] I prefer not to answer
[ ] Does not apply
[ ] Don't know

What country do you live in?*
( ) United States
( ) Mexico
( ) Other country

What ZIP code do you live in?*
____

Do you consider yourself to be male, female, or transgender?*
( ) Male
( ) Female
( ) Transgender
( ) I prefer not to answer
( ) Don't know

Have you <u>ever</u> had vaginal sex (penis in the vagina) or anal sex (penis in the butt) with a woman?*
( ) No
( ) Yes
( ) I prefer not to answer
( ) Don't know

Have you <u>ever</u> had oral sex (mouth on the penis) with a man?*
( ) No
( ) Yes
( ) I prefer not to answer
( ) Don't know

Have you <u>ever</u> had anal sex (penis in the butt) with a man?*
( ) No
( ) Yes
( ) I prefer not to answer
( ) Don't know

Do you consider yourself to be:*
( ) Homosexual or Gay
( ) Heterosexual or Straight
( ) Bisexual
( ) I prefer not to answer
( ) Don't know

Consent

Thank you for your interest in our survey. The video below will give you more information about the survey. Please watch it and indicate below whether you agree to participate.

You may also download the full consent form at the link below the video.

Please note:

1. Your answers are confidential: we don't have any information about who you are beyond the questions you answer.

2. This survey includes some personal questions. You can choose to not answer any questions that make you feel uncomfortable.

If you have any questions or comments you may contact the research staff at amis@emory.edu.

Please indicate whether you agree to participate in the survey. *
( ) I agree to participate in the survey.
( ) I do not agree to participate in the survey.

Demographics

What is the highest level of education you completed?
( ) Never attended school
( ) Less than high school
( ) Some high school
( ) High school diploma or GED
( ) Some college, Associate’s Degree, or Technical Degree
( ) College, post graduate or professional school
( ) I prefer not to answer
( ) Don't know

What was your household income last year from all sources before taxes? That is, the total amount of money earned and shared by all people living in your household.
( ) $0 to $19,999 annually ($0 to $1667 monthly)
( ) $20,000 to $39,999 annually ($1668 to $3333 monthly)
( ) $40,000 to $74,999 annually ($3334 to $6250 monthly)
( ) $75,000 or more annually ($6251 or more monthly)
( ) I prefer not to answer
( ) Don't know

Including yourself, how many people depend on this income?

____

How many of your dependents are under the age of 18?

____

In the past 12 months, did you or other adults in the household ever cut the size of your meals or skip meals because there wasn't enough money for food?
( ) No
( ) Yes
( ) I prefer not to answer
( ) Don't know

How often did this happen?
( ) Almost every month
( ) Some months but not every month
( ) Only 1 or 2 months
( ) I prefer not to answer
( ) Don't know

Housing and Health History

In the past 12 months, did you double up or stay overnight with friends, relatives, or someone you didn’t know well because you didn’t have a regular, adequate, and safe place to stay at night?
( ) No
( ) Yes
( ) I prefer not to answer
( ) Don't know

In the past 12 months, were you ever homeless? That is, were you living on the street, in a shelter, in a Single Room Occupancy hotel (SRO), or in a car?
( ) No
( ) Yes
( ) I prefer not to answer
( ) Don't know

What kind of health insurance or health care coverage do you currently have?
Health insurance--health plans people get through employment or purchased directly as well as government programs (like Medicare and Medicaid) that provide medical care or help pay medical bills.
Choose all that apply:
[ ] A private health plan purchased through an employer
[ ] A private health plan purchased through an exchange (i.e. Obamacare)
[ ] Medicaid or Medicare
[ ] Some other Medical Assistance program
[ ] TRICARE (CHAMPUS)
[ ] Veterans Administration coverage
[ ] Some other health care plan
[ ] I don't currently have any health insurance
[ ] I prefer not to answer
[ ] Don't know

In the past 12 months, have you seen a doctor, nurse, or other health care provider about your own health?
( ) No
( ) Yes
( ) I prefer not to answer
( ) Don't know

At any of those times you were seen by a doctor or health care provider, were you offered an HIV test? An HIV test checks whether someone has the virus that causes AIDS.
( ) No
( ) Yes
( ) I prefer not to answer
( ) Don't know

Did your doctor or health care provider talk to you about sex (gay or straight) or sexual health?
( ) No
( ) Yes
( ) I prefer not to answer
( ) Don't know

Do you consider yourself to be:
( ) Heterosexual or "Straight"
( ) Homosexual or Gay
( ) Bisexual
( ) I prefer not to answer
( ) Don't know

Outness

Have you ever told anyone that you are attracted to or have sex with men?
( ) No
( ) Yes
( ) I prefer not to answer
( ) Don't know

Who of the following people have you told that you are attracted to or have sex with men?

	No	Yes	Does not apply
Gay, lesbian, or bisexual friends	( )	( )	( )
Friends who are not gay, lesbian, or bisexual	( )	( )	( )
Family members	( )	( )	( )
Health care provider	( )	( )	( )
Employer	( )	( )	( )
Fellow employees	( )	( )	( )

Marriage

Have you ever been legally married?
( ) No
( ) Yes

Are you currently legally married?
( ) No
( ) Yes

What is the gender of the partner to whom you are legally married?
( ) Male
( ) Female
( ) Transgender (Male-to-Female)
( ) Transgender (Female-to-Male)

Substance Use: Injection Drug Use

The next questions are about injection drug use. This means injecting drugs yourself or having someone who isn't a health care provider inject you.

Have you ever in your life shot up or injected any drugs other than those prescribed for you? By shooting up, we mean anytime you might have used drugs with a needle, either by mainlining, skin popping, or muscling.

( ) No
( ) Yes
( ) I prefer not to answer
( ) Don't know

In the past 12 months, on average, how often did you inject?

( ) More than once a day
( ) Once a day
( ) More than once a week
( ) Once a week
( ) More than once a month
( ) Once a month
( ) Less than once a month
( ) Never
( ) I prefer not to answer
( ) Don't know

Which drug do you inject most often?

( ) Speedball - Heroin and cocaine together
( ) Heroin, by itself
( ) Cocaine, by itself
( ) Crack
( ) Crystal, meth, tina, crank, ice
( ) Something else (Specify): ____
( ) I prefer not to answer
( ) Don't know

Substance Use: Non-Injection Drug Use

The next questions are about drugs that you may have used but did not inject.

In the past 12 months, have you used any non-injection drugs (drugs you did not inject), other than those prescribed for you.

( ) No
( ) Yes
( ) I prefer not to answer
( ) Don't know

In the past 12 months, which drugs that were not prescribed to you did you use? (Check all that apply.)
[ ] Marijuana
[ ] Powdered cocaine (smoked or snorted)
[ ] Poppers (amyl nitrate)
[ ] X or Ecstasy
[ ] Painkillers (Oxycontin, Vicodin, Percocet)
[ ] Downers (Valium, Ativan, Xanax)
[ ] Crystal meth (tina, crank, ice)
[ ] Hallucinogens (LSD, mushrooms)
[ ] Special K (ketamine)
[ ] GHB
[ ] Crack cocaine
[ ] Other drug: ____
[ ] Heroin (smoked or snorted)
[ ] I prefer not to answer
[ ] Don't know

Substance Use: Drug use frequency

In the past 12 months, how often did you use [question("piped title")]?
( ) More than once a day
( ) Once a day
( ) More than once a week
( ) Once a week
( ) More than once a month
( ) Once a month
( ) Less than once a month
( ) I prefer not to answer
( ) Don't know

Substance Use: Legal Marijuana

In the past 12 months, have you been prescribed marijuana and had it filled at a legal dispensary?
( ) No
( ) Yes
( ) I prefer not to answer
( ) Don't know

Sexual Behavior: Female Sex Partners

The next questions are about having sex with women. For these questions, "having sex" means oral, vaginal, or anal sex. Oral sex means mouth on the vagina or penis; vaginal sex means penis in the vagina; and anal sex means penis in the anus (butt).

In the past 12 months (since [MONTH/YEAR]), what types of sex have you had with a woman? (Check all that apply.)
[ ] Oral sex
[ ] Vaginal sex
[ ] Anal sex
[ ] Some other type of sex
[ ] I have not had any type of sex with a woman in the past 12 months
[ ] I prefer not to answer
[ ] Don't know

Sexual Behavior: Female Sex Partners

These next questions are about the last time you had oral, vaginal or anal sex with a woman.

Was the woman you had sex with that last time a main partner (someone you felt committed to above anyone else) or a casual partner (someone you didn't feel committed to or don't know very well)?
( ) Main sex partner
( ) Casual sex partner
( ) I prefer not to answer
( ) Don't know

When you had sex that last time, did you have either vaginal or anal sex?
( ) No
( ) Yes
( ) I prefer not to answer
( ) Don't know

The last time you had sex with a woman, did you have either vaginal or anal sex <u>without using a condom</u>?
( ) No
( ) Yes
( ) I prefer not to answer
( ) Don't know

The last time you had sex with this partner, did you know her HIV status?
( ) No
( ) Yes
( ) I prefer not to answer

What was her HIV status?
( ) HIV-negative
( ) HIV-positive
( ) Indeterminate
( ) I prefer not to answer

Sexual Behavior: Male Sex Partners

The next questions are about having sex with men. For these questions, "having sex" means oral or anal sex. Oral sex means he put his mouth on your penis or you put your mouth on his penis. Anal sex means you put your penis in his anus (butt) or he put his penis in your anus (butt).

How old were you the first time you had oral sex (mouth on the penis) with a man?
____

How old were you the first time you had anal sex (penis in the butt) with a man?
____

Do you consider yourself to be a top, bottom, or versatile?
( ) Top
( ) Bottom
( ) Versatile
( ) I prefer not to answer
( ) Don't know

Sexual Behavior: Male Sex Partners

In the past 12 months (since [MONTH/YEAR]), what types of sex have you had with other men?
[ ] Oral sex
[ ] Anal sex
[ ] Some other type of sex
[ ] I have not had any type of sex with a man in the past 12 months
[ ] I prefer not to answer
[ ] Don't know

Please specify other type of sex:
____

In the past 12 months (since [MONTH/YEAR]), with how many different men have you had oral or anal sex?
____

In the past 12 months (since [MONTH/YEAR]), with how many different men have you had <u>anal</u> sex?
____

In the past 12 months, since [MONTH/YEAR], with how many different men have you had <u>oral</u> sex?
____

Sexual Behavior: Male Sex Partners, 3

Of the [NUMBER] men you had oral or anal sex with in the past 12 months (since [MONTH/YEAR]), how many of them did you have <u>anal</u> sex with?
____

Sexual Behavior: Condom Use

In the past 12 months (since [MONTH/YEAR]), did you have anal sex <u>without using a condom</u>?
( ) No
( ) Yes
( ) I prefer not to answer
( ) Don't know

Sexual Behavior: Condom Use, 2

In the past 12 months (since [MONTH/YEAR]), with how many of these [NUMBER] male anal sex partners did you have anal sex <u>without using a condom</u>?

____

In the past 12 months (since [MONTH/YEAR]), with how many of these [NUMBER] male anal sex partners did you have anal sex <u>without using a condom</u>?

____

Sexual Behavior: Male Sex Partners (1 Partner)

In the past 12 months (since [MONTH/YEAR]), this male partner was a:
( ) Main partner (someone you felt committed to above anyone else)
( ) Casual partner (someone you didn't feel committed to or don't know very well)
( ) I prefer not to answer
( ) Don't know

Did you know his HIV status?
( ) No
( ) Yes
( ) I prefer not to answer

What was his HIV status?
( ) HIV-negative
( ) HIV-positive
( ) Indeterminate
( ) I prefer not to answer

Sexual Behavior: Male Sex Partners (>1)

In the past 12 months (since [MONTH/YEAR]), the [NUMBER] male partners you told us about were:
( ) Only main partners (you felt committed to above anyone else)
( ) Only casual partners (you didn't feel committed to or don't know very well)
( ) Both main and casual partners
( ) I prefer not to answer
( ) Don't know

In the past 12 months (since [MONTH/YEAR]), did you have anal sex <u>without using a condom</u> with a man whose HIV status you did not know?
( ) No
( ) Yes
( ) I prefer not to answer
( ) Don't know

Was this with a main or casual partner?
( ) Main partner
( ) Casual partner
( ) Both main and casual partners
( ) I prefer not to answer
( ) Don't know

In the past 12 months (since [MONTH/YEAR]) , did you have anal sex <u>without using a condom</u> with a man who was HIV positive?
( ) No
( ) Yes
( ) I prefer not to answer
( ) Don't know

Was this with a main or casual partner?
( ) Main partner
( ) Casual partner
( ) Both main and casual partners
( ) I prefer not to answer
( ) Don't know

In the past 12 months (since [MONTH/YEAR]), did you have anal sex <u>without using a condom</u> with a man who was HIV negative?
( ) No
( ) Yes
( ) I prefer not to answer
( ) Don't know

Was this with a main or casual partner?
( ) Main partner
( ) Casual partner
( ) Both main and casual partners
( ) I prefer not to answer
( ) Don't know

Sexual Behavior: Male Sex Partners (Last Sex)

In the next few screens, we're going to ask some questions about your most recent male sex partner--that is, the last guy you had sex with.

To make the questions easier to ask, we'd like you to enter in this partner's initials or a nickname that is not his real name. If you prefer to leave his initials blank, we will refer to him as "your last partner".

What are the initials of or a nickname for your last male sex partner?
____

Sexual Behavior: Male Sex Partners (Last Sex)

When was the last time you had either oral or anal sex with [INITIALS/NICKNAME]?
Month:
( ) January
( ) February
( ) March
( ) April
( ) May
( ) June
( ) July
( ) August
( ) September
( ) October
( ) November
( ) December
Year:: ____

Was [INITIALS/NICKNAME] a main partner (someone you felt committed to above anyone else) or a casual partner (someone you didn't feel committed to or don't know very well)?
( ) Main sex partner
( ) Casual sex partner
( ) I prefer not to answer
( ) Don't know

Sexual Behavior: Male Sex Partners (Last Sex)

That last time you had sex with [INITIALS/NICKNAME], did you have receptive anal sex where he put his penis in your anus (you were the bottom)?
( ) No
( ) Yes
( ) I prefer not to answer
( ) Don't know

During that last time you had receptive anal sex, did [INITIALS/NICKNAME] use a condom?
( ) No
( ) Yes, but not the whole time
( ) Yes, the whole time
( ) I prefer not to answer
( ) Don't know

When you had sex that last time, did you have insertive anal sex where you put your penis in his anus (you were the top)?
( ) No
( ) Yes
( ) I prefer not to answer
( ) Don't know

During insertive anal sex that last time, did you use a condom?
( ) No
( ) Yes, but not the whole time
( ) Yes, the whole time
( ) I prefer not to answer
( ) Don't know

Sexual Behavior: Male Sex Partners (Last sex HIV)

The last time you had sex with [INITIALS/NICKNAME], did you know his HIV status?
( ) No
( ) Yes
( ) I prefer not to answer

What was [INITIALS/NICKNAME]'s HIV status?
( ) HIV-negative
( ) HIV-positive
( ) Indeterminate
( ) I prefer not to answer

Last sex partner - age

What was [INITIALS/NICKNAME]'s age?
( ) 19 years or younger
( ) 20 to 24 years
( ) 25 to 29 years
( ) 30 to 34 years
( ) 35 to 39 years
( ) 40 to 44 years
( ) 45 to 49 years
( ) 50 to 54 years
( ) 55 to 59 years
( ) 60 to 64 years
( ) 65 to 69 years
( ) 70 to 74 years
( ) 75 to 79 years
( ) 80 years or older

( ) Don't know
( ) Prefer not to answer

Was [INITIALS/NICKNAME] younger than you, older than you, or the same age as you?
[ ] Younger
[ ] Older
[ ] Same age
[ ] I prefer not to answer
[ ] Don't know

Which of the following best describes [INITIALS/NICKNAME]'s ethnic background?
[ ] American Indian or Alaska Native
[ ] Asian
[ ] Black or African American
[ ] Hispanic or Latino
[ ] Native Hawaiian or other Pacific Islander
[ ] White
[ ] I prefer not to answer
[ ] Don't know

As far as you know, has [INITIALS/NICKNAME] ever injected drugs like heroin, cocaine, or speed?

Would you say he:
( ) Definitely did not
( ) Probably did not
( ) Probably did
( ) Definitely did
( ) I prefer not to answer
( ) Don't know

As far as you know, has [INITIALS/NICKNAME] ever used crystal meth (tina, crank, ice)?
( ) Definitely did not
( ) Probably did not
( ) Probably did
( ) Definitely did
( ) I prefer not to answer
( ) Don't know

Did you have sex with [INITIALS/NICKNAME] one time ('one night stand'), or more than one time?
( ) One time
( ) More than one time
( ) I prefer not to answer
( ) Don't know

How long have you been having a sexual relationship with [INITIALS/NICKNAME]?
.: ____
.
( ) Days
( ) Months
( ) Years

Do you expect to have sex with [INITIALS/NICKNAME] again?
( ) No
( ) Yes
( ) I prefer not to answer
( ) Don't know

Sexual Behavior: Male Sex Partners (Last sex relationship)

As far as you know, during the time you were having a sexual relationship with [INITIALS/NICKNAME], did he have sex with other people?

Would you say he:
( ) Definitely did not
( ) Probably did not
( ) Probably did
( ) Definitely did
( ) I prefer not to answer
( ) Don't know

During the time you were having a sexual relationship with [INITIALS/NICKNAME], did you have sex with other people?
( ) No
( ) Yes
( ) I prefer not to answer
( ) Don't know

Sexual Behavior: Male Sex Partners (Last sex relationship)

Where did you first meet [INITIALS/NICKNAME]?
( ) Work
( ) School
( ) House party
( ) Mobile phone app (such as a gay chat, dating or hookup app)
( ) Internet
( ) Bar/Club
( ) Circuit party or rave
( ) Public sex environment (such as a bathhouse, sex club, sex resort, cruising area, private sex party, or adult bookstore)
( ) Place or worship (such as a church, synagogue, mosque)
( ) Other: ____
( ) I prefer not to answer
( ) Don't know

Alcohol and Drugs at last sex

Before or during the last time you had sex with [INITIALS/NICKNAME], did you use:
( ) Alcohol
( ) Drugs
( ) Both alcohol and drugs
( ) Neither one
( ) I prefer not to answer
( ) Don't know

Alcohol and Drugs at last sex

For the next question, a drink of alcohol is a 12 oz beer, a 5 oz glass of wine, or a 1.5 oz shot of liquor. A 40 oz beer would count as 3 drinks. A cocktail with 2 shots would count as 2 drinks.

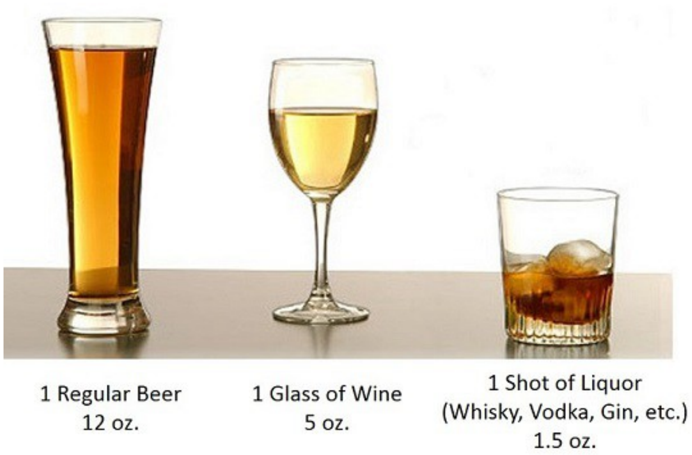

Before or during sex the last time you had sex with [INITIALS/NICKNAME], how many alcoholic drinks did you have?
____

Alcohol and Drugs at last sex

The last time you had sex with [INITIALS/NICKNAME], which drugs did you use? Check all that apply.
[ ] Other drug: ____
[ ] I prefer not to answer
[ ] Don't know

Sexual Behavior: Social Habits

In the past 12 months, have you exchanged things like money or drugs for sex with a male partner? Check all that apply.
[ ] No
[ ] Yes, I gave a sex partner things like drugs or money for sex
[ ] Yes, a sex partner gave me things like drugs or money for sex
[ ] I prefer not to answer
[ ] Don't know

In the past 12 months, how often have you gone to a place (not online) where gay men hangout, meet or socialize? These could include bars, clubs, social organizations, parks, gay businesses, bookstores, sex clubs, etc.
( ) More than once a day
( ) Once a day
( ) More than once a week
( ) Once a week
( ) More than once a month
( ) Once a month
( ) Less than once a month
( ) Never
( ) I prefer not to answer
( ) Don't know

In the past 12 months, have you used any of following kinds of internet sites to meet or socialize with gay men?
[ ] Social network websites (such as Facebook)
[ ] Dating websites directed towards gay men
[ ] Mobile phone apps (such as gay chat, dating and hookup apps)
[ ] None of the above
[ ] I prefer not to answer
[ ] Don't know

In the past 12 months, how often did you use social network websites (such as Facebook) to meet or socialize with gay men?
( ) More than once a day
( ) Once a day
( ) More than once a week
( ) Once a week
( ) More than once a month
( ) Once a month
( ) Less than once a month
( ) Never
( ) I prefer not to answer
( ) Don't know

In the past 12 months, how often have you used dating websites directed towards gay men to meet or socialize with gay men?
( ) More than once a day
( ) Once a day
( ) More than once a week
( ) Once a week
( ) More than once a month
( ) Once a month
( ) Less than once a month
( ) Never
( ) I prefer not to answer
( ) Don't know

In the past 12 months, how often did you use mobile phone apps (such as gay chat, dating and hookup apps) to meet or socialize with gay men?
( ) More than once a day
( ) Once a day
( ) More than once a week
( ) Once a week
( ) More than once a month
( ) Once a month
( ) Less than once a month
( ) Never
( ) I prefer not to answer
( ) Don't know

How satisfied are you with your current sex life?
( ) Very satisfied
( ) Satisfied
( ) Unsure
( ) Dissatisfied
( ) Very Dissatisfied
( ) I prefer not to answer
( ) Don't know

HIV Testing

Have you ever been tested for HIV? An HIV test checks whether someone has the virus that causes AIDS.
( ) No
( ) Yes
( ) I prefer not to answer
( ) Don't know

In the past 2 years (since [MONTH/YEAR]), how many times have you been tested for HIV?

____

When did you have your most recent HIV test?
If you don't know the exact month, please enter your best guess.
Month:
( ) January
( ) February
( ) March
( ) April
( ) May
( ) June
( ) July
( ) August
( ) September
( ) October
( ) November
( ) December
Year:: ____

HIV Testing

Have you had an HIV test in the past 12 months (since [MONTH/YEAR])?
( ) No
( ) Yes
( ) I prefer not to answer
( ) Don't know

When you got tested in [MONTH/YEAR OF LAST HIV TEST] where did you get tested?
( ) Private doctor's office
( ) HIV counseling and testing site
( ) Public health clinic/community health clinic
( ) Street outreach program/mobile unit
( ) Sexually transmitted disease clinic
( ) Hospital (inpatient)
( ) Correctional facility (jail or prison)
( ) Emergency room
( ) At home
( ) Other
( ) I prefer not to answer
( ) Don't know

HIV Status

What was the result of your most recent HIV test?
( ) Negative
( ) Positive
( ) Never obtained results
( ) Indeterminate
( ) I prefer not to answer
( ) Don't know

Before your test in [MONTH/YEAR OF LAST HIV TEST], did you <u>ever</u> test positive for HIV?
( ) No
( ) Yes
( ) I prefer not to answer
( ) Don't know

Was your test in [MONTH/YEAR OF LAST HIV TEST] your first positive test?
( ) No
( ) Yes
( ) I prefer not to answer
( ) Don't know

When did you first test positive?

Month
( ) January
( ) February
( ) March
( ) April
( ) May
( ) June
( ) July
( ) August
( ) September
( ) October
( ) November
( ) December
Year: ____

Are you currently taking antiretroviral medicines to treat your HIV infection?
( ) No
( ) Yes
( ) I prefer not to answer
( ) Don't know

What is the main reason you are not currently taking any antiretroviral medicines?

( ) Not currently going to a health care provider for my HIV
( ) CD4 count and viral load are good
( ) Don't have money or insurance for antiretroviral medicines
( ) Don't want to take antiretroviral medicines
( ) Other
( ) I prefer not to answer
( ) Don't know

At-home HIV Testing

A <u>home HIV test</u> is one that you can use to test yourself for HIV at home or another private location.

Have you ever heard about home HIV tests?
( ) No
( ) Yes
( ) I prefer not to answer

Have you ever used a home HIV test?
( ) No
( ) Yes
( ) I prefer not to answer

In the past 12 months (since [MONTH/YEAR]), how many times have you tested yourself with a home HIV test?
____

In the past 12 months (since [MONTH/YEAR]), which of these home HIV tests have you used? Check all that apply.
[ ] Home Access HIV- Test System (where you prick your finger, collect a blood sample on a card, and mail that card to a lab for testing)
[ ] Oraquick In-Home HIV Test (where you collect your own oral fluid sample by swabbing your mouth, use the testing device yourself, and read the test results within 20 minutes)
[ ] Other rapid HIV test (please specify):
____
[ ] I prefer not to answer
[ ] Don't know

Under which circumstances did you use the home HIV test? Check all that apply.
[ ] I used it to test myself regularly
[ ] I used it to test myself before having sex with a new partner
[ ] I asked my sex partner to test himself/herself before having sex with me
[ ] I used it to test myself after having sex with someone I knew was HIV negative
[ ] I used it to test myself after having sex with someone I knew was HIV positive OR whose HIV status I did not know
[ ] I prefer not to answer
[ ] Don't know
[ ] Other: ____

Home Testing Questions (continued)

You mentioned you used [NAME OF TEST] to test yourself for HIV. Where did you get the home HIV test(s)? Check all that apply.
[ ] Bought Online
[ ] From a pharmacy
[ ] From a friend
[ ] From a sex partner
[ ] From my doctor’s office
[ ] From an HIV counseling and testing site
[ ] From a Health Department
[ ] From another research study
[ ] Other location or person (Specify (do not include someone’s name))
[ ] I prefer not to answer
[ ] Don't know

HIV Testing Reasons

Think about the last time you had anal sex without a condom with a man you didn’t feel committed to or don’t know very well (that is, a casual sex partner). Did you get tested for HIV because of this sex act?

( ) No
( ) Yes
( ) I’ve never had anal sex without a condom with a casual sex partner
( ) I prefer not to answer
( ) Don't know

Thinking about the last time you had anal sex without a condom with a man who you knew was HIV-positive. Did you get tested for HIV because of this sex act?

( ) No
( ) Yes
( ) I've never had anal sex without a condom with an HIV-positive partner
( ) I prefer not to answer

( ) Don't know

Think about the last time you started a new relationship with someone you felt committed to above anyone else, like a boyfriend, spouse, or main partner. Did you get tested for HIV because of this new relationship?

( ) No
( ) Yes
( ) I've never been in a relationship like that
( ) I prefer not to answer
( ) Don't know

You mentioned having been tested for HIV in the past 12 months. The last time you got tested for HIV, did you get tested in response to any of the following events?

( ) Having anal sex without a condom with a man you didn’t feel committed to or didn’t know very well (a casual sex partner)
( ) Having anal sex without a condom with a man you knew to be HIV-positive
( ) Starting a new relationship with a main sex partner (someone you felt committed to above anyone else)
( ) I tested for a different reason
( ) I prefer not answer
( ) Don't know

HIV Testing Reasons, CONTD

How many weeks after having anal sex without a condom with a casual sex partner did you get tested for HIV? By casual partner, we mean someone you didn't feel committed to or didn't know very well.

____

How many weeks after having anal sex without a condom with a man you knew to be HIV positive did you get tested for HIV?

____

How many weeks after starting a new relationship with a main sex partner did you get tested for HIV? By main partner, we mean someone you felt committed to above anyone else.

____

PrEP Module

Pre-exposure prophylaxis (PrEP) is taking an antiretroviral pill, also called Truvada, every day for months or years to reduce a persons chance of getting HIV.

Before today, have you ever heard of people who do not have HIV taking PrEP, the antiretroviral medicine taken every day for months or years to reduce the risk of getting HIV?
( ) No
( ) Yes

In the past 12 months (since [MONTH/YEAR]), have you had a discussion with a health care provider about taking PrEP?
( ) No
( ) Yes
( ) I prefer not to answer
( ) Don't know

In the past 12 months (since [MONTH/YEAR]), when you discussed taking PrEP with a health care provider, did you receive the medicine or a prescription for the medicine?
( ) No
( ) Yes
( ) I prefer not to answer
( ) Don't know

Have you ever taken PrEP?
( ) No
( ) Yes
( ) I prefer not to answer
( ) Don't know

Are you currently taking PrEP?
( ) No
( ) Yes
( ) I prefer not to answer
( ) Don't know

In the last 30 days, about how many doses of PrEP did you take?
( ) Less than 15
( ) 16-29
( ) 30

The last time you were on PrEP, how many months in a row were you taking it?
( ) Less than 2 months
( ) 2 to 6 months
( ) 7 to 12 months
( ) I prefer not to answer
( ) Don't know

PrEP, continued

PrEP stands for pre-exposure prophylaxis. It involves a healthy person taking a pill used to treat HIV in order to prevent being infected with HIV. The pills have to be taken once a day, every day. Some people who take these pills experience side effects. These may include nausea and weight loss, which usually go away after the first month. In rare cases, taking the pill for long periods may damage the kidneys. The medication is prescribed by a doctor. Taking this medication provides only partial protection against HIV infection. So, a person on the medication should still practice other HIV prevention strategies like using condoms every time. For people who take the pill every day, studies have shown that it provides up to 90% protection against HIV infection.

Would you be willing to take anti-HIV medicines every day to lower your chances of getting HIV?
( ) No
( ) Yes
( ) I prefer not to answer
( ) Don't know

STD Concern

How concerned are you about becoming infected with a sexually transmitted disease other than HIV (i.e., syphilis, chlamydia, gonorrhea)?

( ) Very concerned
( ) Somewhat concerned
( ) Neither concerned nor unconcerned
( ) Somewhat unconcerned
( ) Not concerned at all
( ) Don't know

STD-PrEP

Pre-exposure prophylaxis, or PrEP, has been shown to reduce the risk of acquisition of HIV but is not designed for prevention of other sexually transmitted diseases (STDs, like syphilis, chlamydia, or gonorrhea). Researchers are exploring the development of an "STD-PrEP" which could be a daily pill or a monthly injection given by a doctor.

How likely would you be to use "STD-PrEP" as a <u>daily pill</u> were it available?
( ) Very likely
( ) Somewhat likely
( ) Neither likely nor unlikely
( ) Somewhat unlikely
( ) Very unlikely
( ) Don't know

How likely would you be to use "STD-PrEP" as a <u>monthly injection given by a doctor</u>, were it available?
( ) Very likely
( ) Somewhat likely
( ) Neither likely nor unlikely
( ) Somewhat unlikely
( ) Very unlikely
( ) Don't know

PEP

<u>Post-exposure prophylaxis (PEP)</u> is taking antiretroviral pills for 28 days after a person has had a single high-risk exposure to HIV.

In the past 12 months (since [MONTH/YEAR]), have you taken PEP to reduce the risk of getting HIV?
( ) No
( ) Yes
( ) Don't know
( ) I prefer not to answer

Stigma - NHBS Measure

In the past 12 months, have any of the following things happened to you because someone knew or assumed you were attracted to men?

	Yes	No	I prefer not to answer	Don't know	Does not apply
You were called names or insulted	( )	( )	( )	( )	( )
You received poorer services than other people in restaurants, stores, other businesses or agencies	( )	( )	( )	( )	( )
You were treated unfairly at work or school	( )	( )	( )	( )	( )
You were denied or given lower quality health care	( )	( )	( )	( )	( )
You were physically attacked or injured	( )	( )	( )	( )	( )

How much do you agree or disagree with the following:

Most people in my area are tolerant of gays and bisexuals.
( ) Strongly agree
( ) Agree
( ) Neither agree or disagree
( ) Disagree
( ) Strongly disagree
( ) I prefer not to answer
( ) Don't know

Viral STI Diagnoses

Has a doctor, nurse or other health care provider ever told you that you had any of the following? Check all that apply.
[ ] Hepatitis
[ ] Genital herpes
[ ] Genital warts
[ ] Human papillomavirus or HPV
[ ] None of the above
[ ] I prefer not to answer
[ ] Don't know

What type or types of hepatitis have you had? Check all that apply.
[ ] Hepatitis A
[ ] Hepatitis B
[ ] Hepatitis C
[ ] Other
[ ] I prefer not to answer
[ ] Don't know

There are vaccines or shots that can prevent some types of hepatitis. Have you ever had a hepatitis vaccine?
( ) No
( ) Yes
( ) I prefer not to answer
( ) Don't know

What type or types of hepatitis vaccine have you had?
( ) Hepatitis A vaccine
( ) Hepatitis B vaccine
( ) Both Hepatitis A and B vaccine
( ) I prefer not to answer
( ) Don't know

Bacterial STI Diagnoses

In the past 12 months (since [MONTH/YEAR]), has a doctor, nurse or other health care provider told you that you had any of the following? Check all that apply.
[ ] Gonorrhea
[ ] Chlamydia
[ ] Syphilis
[ ] None of the above
[ ] I prefer not to answer
[ ] Don't know

Bacterial STI Testing

Even though a doctor, nurse, or other health care provider did not tell you that you had gonorrhea, in the past 12 months (since [MONTH/YEAR]) were you tested for gonorrhea?
( ) No
( ) Yes
( ) I prefer not to answer
( ) Don't know

Even though a doctor, nurse, or other health care provider did not tell you that you had chlamydia, in the past 12 months (since [MONTH/YEAR]) were you tested for chlamydia?
( ) No
( ) Yes
( ) I prefer not to answer
( ) Don't know

Even though a doctor, nurse, or other health care provider did not tell you that you had syphilis, in the past 12 months (since [MONTH/YEAR]) were you tested for syphilis?
( ) No
( ) Yes
( ) I prefer not to answer
( ) Don't know

Rectal STI screening

In the past 12 months (since [MONTH/YEAR]), have you been tested for rectal gonorrhea? This usually involves collection of a rectal (butt) swab either by you or a health care professional.
( ) No
( ) Yes
( ) I prefer not to answer
( ) Don't know

In the past 12 months (since [MONTH/YEAR]), have you been tested for rectal chlamydia? This usually involves collection of a rectal (butt) swab either by you or a health care professional.
( ) No
( ) Yes
( ) I prefer not to answer
( ) Don't know

HPV

A vaccine to prevent human papillomavirus (HPV) infection is available and is called the HPV shot, cervical cancer vaccine, GARDASIL®, or CERVARIX®. Have you ever received the HPV vaccine?
( ) No
( ) Yes
( ) I prefer not to answer
( ) Don't know

How old were you when you received your first dose of the HPV vaccine?
____

Assessment of Prevention Activities

In the past 12 months, have you gotten any free condoms, not counting those given to you by a friend, relative, or sex partner?
( ) No
( ) Yes
( ) I prefer not to answer
( ) Don't know

In the past 12 months, have you had a one-on-one conversation with an outreach worker, counselor, or prevention program worker about ways to prevent HIV? Don't count the times where you had a conversation as part of an HIV test.
( ) No
( ) Yes
( ) I prefer not to answer
( ) Don't know

In the past 12 months, have you been a participant in any organized session(s) involving a small group of people to discuss ways to prevent HIV? Don't include discussions you had with a group of friends.
( ) No
( ) Yes
( ) I prefer not to know
( ) Don't know

Prevention Campaigns

In the past 12 months, how often did you see or hear the following slogans or messages?

Let's stop HIV together:
( ) Never
( ) Rarely
( ) Sometimes
( ) Often
( ) Very Often
( ) I prefer not to answer
( ) Don't know

On a scale of 0 to 5, where 0 means "not very effective" and 5 means "very effective", how effective do you think this slogan or message is?
( ) 0 (Not Effective)
( ) 1
( ) 2
( ) 3
( ) 4
( ) 5 (Very Effective)

Prevention Campaigns

In the past 12 months, how often did you see or hear the following slogans or messages?

Protest:
( ) Never
( ) Rarely
( ) Sometimes
( ) Often
( ) Very Often
( ) I prefer not to answer
( ) Don't know

On a scale of 0 to 5, where 0 means "not very effective" and 5 means "very effective", how effective do you think this slogan or message is?
( ) 0 (Not Effective)
( ) 1
( ) 2
( ) 3
( ) 4
( ) 5 (Very Effective)

Prevention Campaigns

In the past 12 months, how often did you see or hear the following slogans or messages?

ACT Against AIDS:
( ) Never
( ) Rarely
( ) Sometimes
( ) Often
( ) Very Often
( ) I prefer not to answer
( ) Don't know

On a scale of 0 to 5, where 0 means "not very effective" and 5 means "very effective", how effective do you think this slogan or message is?
( ) 0 (Not Effective)
( ) 1
( ) 2
( ) 3
( ) 4
( ) 5 (Very Effective)

Prevention Campaigns

In the past 12 months, how often did you see or hear the following slogans or messages?

Greater than AIDS:
( ) Never
( ) Rarely
( ) Sometimes
( ) Often
( ) Very Often
( ) I prefer not to answer
( ) Don't know

On a scale of 0 to 5, where 0 means "not very effective" and 5 means "very effective", how effective do you think this slogan or message is?
( ) 0 (Not Effective)
( ) 1
( ) 2
( ) 3
( ) 4
( ) 5 (Very Effective)

Prevention Campaigns

In the past 12 months, how often did you see or hear the following slogans or messages?

Start Talking. Stop HIV.
( ) Never
( ) Rarely
( ) Sometimes
( ) Often
( ) Very Often
( ) I prefer not to answer
( ) Don't know

On a scale of 0 to 5, where 0 means "not very effective" and 5 means "very effective", how effective do you think this slogan or message is?
( ) 0 (Not Effective)
( ) 1
( ) 2
( ) 3

( ) 4
( ) 5 (Very Effective)

Prevention Campaigns

In the past 12 months, how often did you see or hear the following slogans or messages?

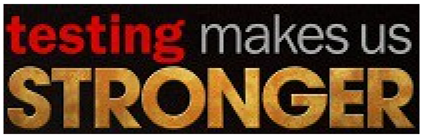

Testing Makes us Stronger:
( ) Never
( ) Rarely
( ) Sometimes
( ) Often
( ) Very Often
( ) I prefer not to answer
( ) Don't know

On a scale of 0 to 5, where 0 means "not very effective" and 5 means "very effective", how effective do you think this slogan or message is?
( ) 0 (Not Effective)
( ) 1
( ) 2
( ) 3
( ) 4
( ) 5 (Very Effective)

Prevention Campaigns

In the past 12 months, how often did you see or hear the following slogans or messages?

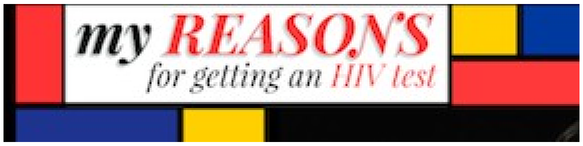

Reasons:
( ) Never
( ) Rarely
( ) Sometimes
( ) Often

( ) Very Often
( ) I prefer not to answer
( ) Don't know

On a scale of 0 to 5, where 0 means "not very effective" and 5 means "very effective", how effective do you think this slogan or message is?
( ) 0 (Not Effective)
( ) 1
( ) 2
( ) 3
( ) 4
( ) 5 (Very Effective)

Prevention Campaigns

In the past 12 months, how often did you see or hear the following slogans or messages?

HIV Treatment Works:
( ) Never
( ) Rarely
( ) Sometimes
( ) Often
( ) Very Often
( ) I prefer not to answer
( ) Don't know

On a scale of 0 to 5, where 0 means "not very effective" and 5 means "very effective", how effective do you think this slogan or message is?
( ) 0 (Not Effective)
( ) 1
( ) 2
( ) 3
( ) 4
( ) 5 (Very Effective)

Study Target Inquiries

As far as you know, did you participate in the "Sex is the Question" Survey between September 2015 and April 2016?
( ) Yes
( ) No
( ) I'm not sure

For this national study, we are recruiting a large number of men like you. Can you tell us the name of social networking website where we could reach other men like you who might like to complete this survey?
____

Future Contact

The PRISM Health team conducts many research projects at Emory University. Would you like to be contacted for potential participation in our future projects?
( ) Yes
( ) No

Please provide the email address you would like for us to use to contact you for future studies.
____

Survey End

Thank you for taking our survey! Your response is very important to us!

If you have any questions or comments, you may contact study staff at amis@emory.edu.

To get more information about HIV, please visit: www.cdc.gov/hiv

Otherwise, you can close your browser.
